# Preoperative radiation may improve the outcomes of resectable IIIA/N2 non‐small‐cell lung cancer patients: A propensity score matching‐based analysis from surveillance, epidemiology, and end results database

**DOI:** 10.1002/cam4.1701

**Published:** 2018-07-29

**Authors:** Dawei Chen, Haiyong Wang, Xinyu Song, Jinbo Yue, Jinming Yu

**Affiliations:** ^1^ Department of Radiotherapy Shandong Cancer Hospital affiliated to Shandong University Jinan Shandong China; ^2^ Shandong Cancer Hospital and Institute Shandong Academy of Medical Sciences Shandong Cancer Hospital affiliated to Shandong University Jinan Shandong China; ^3^ School of Medicine and Life Sciences University of Jinan‐Shandong Academy of Medical Sciences Jinan Shandong China

**Keywords:** IIIA/N2, non‐small cell lung cancer, preoperative radiation, prognosis, propensity score matching

## Abstract

**Background:**

There are several therapeutic strategies for the management of resectable stage IIIA/N2 non‐small‐cell lung cancer (NSCLC) patients. However, the role of radiotherapy as a preoperative adjuvant therapy is unclear.

**Methods:**

We retrospectively analyzed the data of stage IIIA/N2 NSCLC patients who either underwent preoperative radiation (PrORT), or did not undergo preoperative radiation, collected from the Surveillance, Epidemiology and End Results (SEER) database, between 2004 and 2013. The primary endpoints were cancer‐specific survival (CSS) and overall survival (OS).

**Results:**

Ultimately, 493 patients treated with preoperative radiation and 2675 patients treated who were not treated with preoperative radiation, were included in the analysis. Overall, preoperative radiation was associated with a better CSS (HR: 1.427 [1.297‐1.572], *P* = 0.014) and OS (HR: 1.220 [1.131‐1.493], *P* = 0.002) than that observed in patients who did not undergo preoperative radiation. After PSM, preoperative radiation still showed advantage in both CSS and OS. Only age, T stage, and preoperative radiation remained independent prognostic factors for both OS and CSS. In the subgroup analysis, the advantages of preoperative radiotherapy were more pronounced in patients with stage T3 tumors and highly differentiated tumors.

**Conclusions:**

Preoperative radiation may improve the outcomes of resectable IIIA/N2 NSCLC patients. In IIIA/N2 NSCLC patients, particularly with T3 and highly differentiated tumors, clinicians should boldly apply preoperative radiotherapy to improve the patients' survival.

## 1 INTRODUCTION

The treatment for potentially resectable stage IIIA/N2 non‐small‐cell lung carcinoma (NSCLC) remains controversial. Currently, there are several combined modality approaches that are preferred, including surgery after induction chemotherapy, surgery after accelerated radiotherapy following chemotherapy, palliative radiotherapy, surgery after induced chemoradiation, and chemotherapy after induction of synchronous surgery.^1^, ^2^, ^3^, ^4^ However, it is unclear which of these is the optimal modality as the 5‐year survival rates of patients receiving any of these treatments are 20%‐45%.^5^ It is even more difficult to determine the best treatment regimen because stage III NSCLC patients form a very broad population, particularly patients with stage IIIA/N2 tumors.^6^ This is because the tumor size, lymph node involvement, and comorbidities vary widely. At this point, it appears unwise to decide on a universal treatment plan.

Surgery is often the first choice for such patients, but other preoperative neoadjuvant treatment options are available.^7^ The utilization of radiation before surgery is always debated and many studies report that preoperative chemotherapy alone is sufficient prior to surgery in IIIA‐N2 patients.^8^, ^9^, ^10^, ^11^ On the other hand, some studies have confirmed that preoperative radiotherapy, at a high or standard dose, can significantly improve survival.^12^, ^13^, ^14^, ^15^ In short, the role of radiotherapy as a preoperative adjuvant therapy is unclear. In this study, we sought to answer the question of whether preoperative radiation should be performed in patients with resectable stage IIIA/N2 NSCLC.

## 2 MATERIALS AND METHODS

### 2.1 Patient selection

This retrospective study was conducted by acquiring data from the Surveillance Epidemiology and End Results (SEER) database.^15^, ^16^ Data were obtained by SEER*STAT 8.3.2, in October 2017. Using this software, we screened NSCLC patients between 2004 and 2013. To be included in the study, patients had to meet the following criteria: diagnosis confirmed microscopically, age recorded, active follow‐up, no distant metastasis, and the presence of only one primary tumor. Patients with incomplete staging, of unknown age, unknown cause of death, unknown survival period, and death within 30 days after surgery were excluded.

### 2.2 Ethics statement

This study was mainly based on the SEER database and was conducted in compliance with the Declaration of Helsinki. We obtained permission to access the files of SEER program research data (reference number 11561‐Nov 2016). Informed consent was not required because patients were not personally identified. This study was approved by the Ethics Review Committee of the Shandong Cancer Hospital, affiliated with the Shandong University.

### 2.3 Statistical analysis

For all patients, the following variables were analyzed as follows: age, race, sex, AJCC stage, pathological grade, and history of preoperative radiation. The primary endpoints of this study were cancer‐specific survival (CSS) and overall survival (OS), which were extracted from the SEER database. Baseline characteristics of different groups were compared using chi‐square tests. Survival curves were generated with the use of Kaplan‐Meier estimates. The differences between the curves were analyzed through the Log Rank test. Propensity score matching analysis was used for the matching of patients. Univariate and multivariate COX proportional hazards regression models were utilized to evaluate risk of mortality and conduct subgroup analyses. All statistical tests were two‐sided and results were considered statistically significant when a test of a *P* < 0.05 was obtained. The statistical software SPSS 18.0 (SPSS, IL, Chicago) was used for all data analyses.

## 3 RESULTS

### 3.1 Patient demographics

In total, 493 patients who underwent preoperative radiotherapy (PrORT) and 2675 who did not undergo preoperative radiotherapy (no‐PrORT) were included in this retrospective cohort study. Obvious differences in age, gender, degree of differentiation, pathological type, and T stage, were noted between the two groups (Table 1). Specifically, the PrORT group had a higher proportion of elderly patients, male patients, and grade III‐IV, squamous, T3 tumors. This indicated that the baseline characteristics of the two groups were not balanced. After the 1:1 Propensity Score Matching (PSM), a total of 465 pairs were matched successfully (Table 2). In the final analysis model, baseline characteristics, including age, race, sex, grade of differentiation, pathological findings, and T stage were all balanced.

**Table 1:** Baseline characteristics for patients with PrORT/no‐PrORT before PSM

Variables	Radiation (%)	None (%)	*P*
Age			<0.001
<65	306 (62.1)	1078 (40.3)	
≥65	187 (37.9)	1597 (59.7)	
Race			0.457
White	408 (82.8)	2203 (82.4)	
Black	53 (10.8)	260 (9.7)	
Other	32 (6.5)	212 (7.9)	
Sex			0.022
Male	272 (55.2)	1326 (49.6)	
Female	221 (44.8)	1349 (50.4)	
Grade			<0.001
I‐II	171 (34.7)	1293 (48.3)	
III‐IV	322 (65.3)	1382 (51.7)	
Pathology			0.003
Squamous	149 (30.2)	658 (24.6)	
Adenocarcinoma	232 (47.1)	1478 (55.3)	
Others	112 (22.7)	539 (20.1)	
T stage			<0.001
T1	99 (20.1)	775 (29.0)	
T2	302 (61.3)	1695 (63.4)	
T3	92 (18.7)	205 (7.7)	

**Table 2:** Baseline characteristics for patients with PrORT/no‐PrORT after PSM

Variables	Radiation	None	*P*
Age			1.00
<65	283 (60.9)	283 (60.9)	
≥65	182 (39.1)	182 (39.1)	
Race			1.00
White	389 (83.7)	389 (83.7)	
Black	48 (10.3)	48 (10.3)	
Other	28 (6.0)	28 (6.0)	
Sex			1.00
Male	258 (55.5)	258 (55.5)	
Female	207 (44.5)	207 (44.5)	
Grade			1.00
I‐II	161 (34.6)	161 (34.6)	
III‐IV	304 (65.4)	304 (65.4)	
Pathology			1.00
Squamous	141 (30.3)	141 (30.3)	
Adenocarcinoma	220 (47.3)	220 (47.3)	
Others	104 (22.4)	104 (22.4)	
T stage			1.00
T1	95 (20.4)	95 (20.4)	
T2	297 (63.9)	297 (63.9)	
T3	73 (15.7)	73 (15.7)	

### 3.2 Outcomes of patients before and after PSM

**Figure 1:**
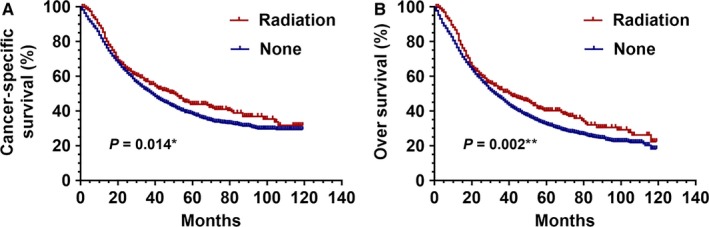
Kaplan‐Meier curves for CSS and OS before PSM. Cancer‐specific survival (A) and overall survival difference (B) between preoperative radiation and no preoperative radiation groups, before 1:1 Propensity Score Matching analysis. *P =* *0.014 for PrORT group compared to no‐PrORT group for CSS; **P =* *0.002 for PrORT group compared to no‐PrORT group for OS

**Figure 2:**
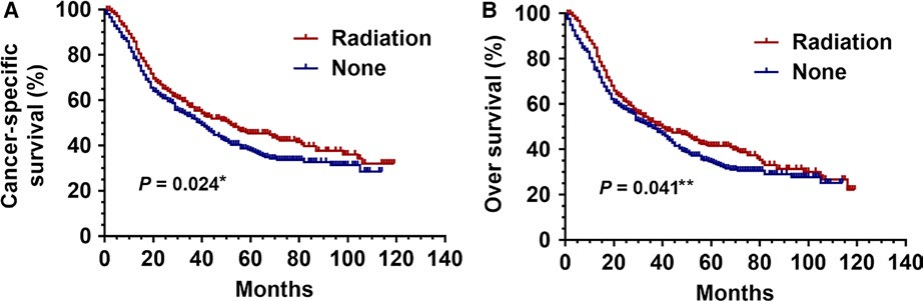
Kaplan‐Meier curves for CSS and OS after PSM. Cancer‐specific survival (A) and overall survival difference (B) between preoperative radiation and no preoperative radiation groups, after 1:1 Propensity Score Matching analysis. *P =* *0.024 for PrORT group compared to no‐PrORT group for CSS; **P =* *0.041 for PrORT group compared to no‐PrORT group for OS

Before PSM, the PrORT group was superior to the no‐PrORT group in both CSS (HR: 1.427 [1.297‐1.572], *P* = 0.014) and OS (HR: 1.220 [1.131‐1.493], *P* = 0.002; Figure 1). The 5‐year survival rates of the PrORT and no‐PrORT groups were 40.9% and 33.8%, respectively (*P* = 0.018). After PSM, OS and CSS for the PrORT group and the no‐PrORT group were similar (Figure 2). A 5‐year survival rate of 43.6% was found in the PrORT group, and 35.2% in the no‐PrORT group (*P* = 0.009).

### 3.3 Univariate and multivariate analysis

In the COX hazard analysis, after testing the proportional hazards assumptions, we selected multiple variables, including age, race, sex, grade, pathology, and T stage. According to the result of univariate COX regression analysis, age, race, sex, grade, pathology, T stage, and radiation were associated with a shorter OS and CSS, after PSM. In the multivariate analysis of CSS and OS, age, T stage, and preoperative radiotherapy were statistically significant (*P* < 0.05). Results of the univariate and multivariate COX regression of prognostic factors for CSS and OS in stage IIIA (cN2) NSCLC patients are shown in Table 3.

**Table 3:** CSS univariate and multivariate Cox regression after PSM

Variables	CSS		
	Univariate	Multivariate	
	*P*	HR (95% CI)	*P*
Age	0.002		0.001
<65		Reference	
≥65		1.388 (1.147‐1.680)	0.001
Race	0.096	Not entered	
White			
Black			
Other			
Sex	0.025		0.065
Female		Reference	
Male		1.196 (0.989‐1.447)	0.065
Grade	0.121	Not entered	
I			
II			
III			
IV			
Pathology	0.046	0.080	
Adenocarcinoma		Reference	
Squamous		1.101 (0.878‐1.380)	0.403
Others		1.304 (1.035‐1.643)	0.024
T stage	0.001		0.001
T1		Reference	
T2		1.189 (0.930‐1.522)	0.168
T3		1.798 (1.314‐2.460)	<0.001
Radiation	0.020		0.010
No		Reference	
Yes		0.783 (0.649‐0.944)	0.010

### 3.4 Subgroup analysis for OS and CSS after PSM

**Figure 3:**
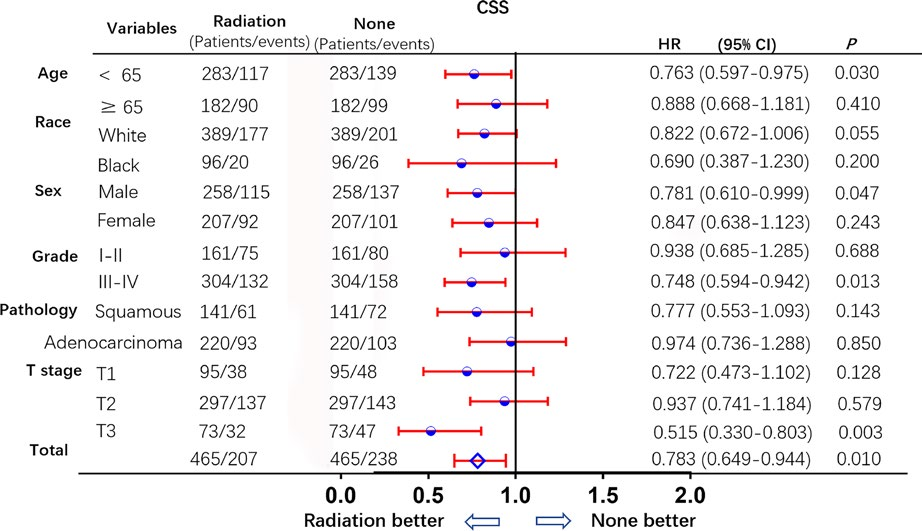
Subgroup analysis for CSS after PSM. Subgroup analysis of cancer‐specific survival between preoperative radiation and no preoperative radiation groups, after 1:1 Propensity Score Matching analysis. All subgroups derived CSS benefit in favor of preoperative radiation group. Age < 65, male, grade III‐IV and T3 were statistically in favor of preoperative radiation group

**Figure 4:**
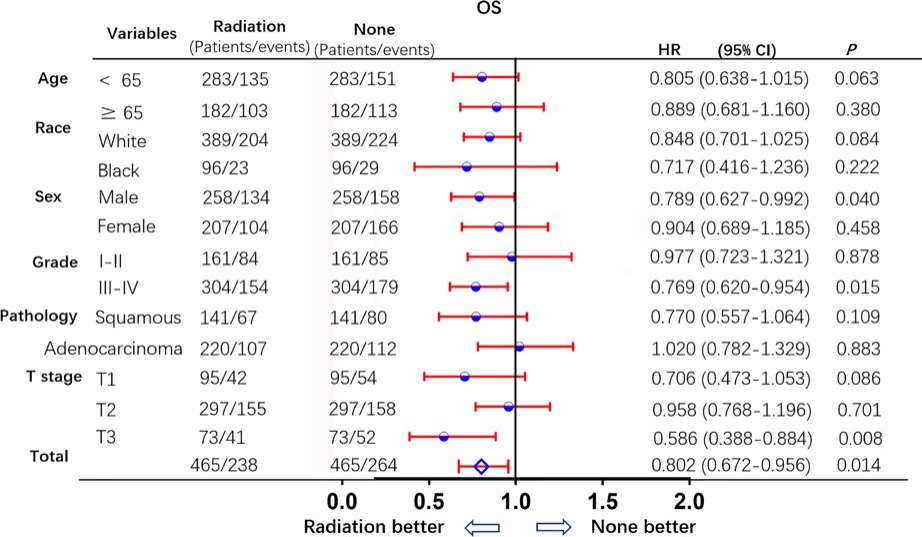
Subgroup analysis for OS after PSM. Subgroup analysis of overall survival between preoperative radiation and no preoperative radiation groups, after 1:1 Propensity Score Matching analysis. All subgroups derived OS benefit statistically in favor of preoperative radiation group, particularly, males, with grade III‐IV and T3 tumors

In the subgroup analysis following PSM, the OS and CSS of patients who underwent PrORT were higher than those of patients in the no‐PrORT group. All subgroups analysis‐derived CSS and OS were in favor of PrORT, as seen in the overall study population (Figures 3 and 4). Age < 65 (HR: 0.763 [0.597‐0.795], *P* = 0.03), males (HR: 0.781 [0.61‐0.999], *P* = 0.047), tumor grade III‐IV (HR: 0.748 [0.594‐0.942], *P* = 0.013), and T3 tumor (HR: 0.515 [0.33‐0.803], *P* = 0.01), were statistically in favor of PrORT in terms of CSS. On the other hand, males (HR: 0.789 [0.627‐0. 992], *P* = 0.04), tumor grade III‐IV (HR: 0.769 [0.62‐0.954], *P* = 0.015) and T3 tumor (HR: 0.586 [0.388‐0.884], *P* = 0.008) were statistically in favor of PrORT, in terms of OS (Table 4).

**Table 4:** OS univariate and multivariate Cox regression after PSM

Variables	OS		
	Univariate	Multivariate	
	*P*	HR (95% CI)	*P*
Age	<0.001		<0.001
<65		Reference	
≥65		1.460 (1.215‐1.753)	<0.001
Race	0.017		0.026
White		Reference	
Black		1.183 (0.877‐1.596)	0.270
Other		0.592 (0.384‐0.913)	0.018
Sex	0.003		0.006
Female		Reference	
Male		1.288 (1.074‐1.545)	0.006
Grade	0.030		0.008
I		Reference	
II		1.526(0.931‐2.501)	0.094
III		1.472 (0.909‐2.383)	0.116
IV		3.697(1.731‐7.896)	0.001
Pathology	0.047	0.013	
Adenocarcinoma		Reference	
Squamous		1.028 (0.827‐1.278)	0.805
Others		1.378 (1.103‐1.721)	0.005
T stage	<0.001		<0.001
T1		Reference	
T2		1.230 (0.973‐1.555)	0.083
T3		1.973 (1.467‐2.655)	<0.001
Radiation	0.035		0.014
No		Reference	
Yes		0.802 (0.672‐0.956)	0.014

## 4 DISCUSSION

More than one‐third of NSCLC patients presented with locally advanced tumors, without distant metastatic disease, which could not be primarily resected.^16^ Radiotherapy is usually used as the primary local treatment but has associated unsatisfying survival rates.^17^, ^18^, ^19^, ^20^ Locally recurrent tumors and their complications account for about 75% of the deaths in patients with epidermoid lung cancer, and approximately 40% of the deaths in patients with other histologic subtypes of NSCLC.^21^, ^22^ To have an impact on the overall outcome of the management of patients with localized NSCLC, one should make an attempt to decrease the probability of local failure as well as distant metastasis.

The rationale for preoperative radiation is to increase the resectability of the tumor, reduce the number of cells capable of implantation at the time of surgery, and to sterilize the microscopic disease.^23^ Several studies^24^, ^25^, ^26^ have demonstrated high resectability rates, ranging from 50% to 72%, with the usage of preoperative radiation. Although the first preoperative trial of radiotherapy in NSCLC by Bromley et al^27^ failed to show an improvement in survival, it did demonstrate that 47% of the patients had no histologically demonstrable tumor in the surgical specimen. Depending on the total dose of radiation delivered preoperatively, other authors^24^, ^26^, ^27^, ^28^, ^29^, ^30^, ^31^ have reported somewhere between 20% and 54% of patients having no persistent tumor or only having microscopic disease.

The results of a phase III, multicenter, randomized clinical trial by the Japan Western Thoracic Cancer Cooperative Group (WJTOC) suggested that the addition of radiotherapy to neoadjuvant treatment may improve the rate of efficacy and stage decline.^15^ In this study, 60 stage III operable NSCLC patients, with a pathological stage of pN2, were randomly divided into neoadjuvant chemotherapy group and neoadjuvant chemoradiation group. The results showed that preoperative neoadjuvant chemoradiotherapy group showed a significant decrease in stage reduction rate (40% and 21%). Median progression‐free survival (PFS, 55.0 months vs 9.4 months) and median OS (63.3 months vs 29.5 months) were significantly greater in patients with mediastinal lymph node stage declining after treatment than in undecreased patients. Therefore, the WJTOC9033 study suggested that adding radiotherapy to induction therapy can improve the local control rate of the tumor without increasing the treatment‐related adverse reactions.

Several prospective randomized studies have failed to demonstrate ant advantages in delivering preoperative radiation, in patients with N2 NSCLC.^26^, ^30^ The reasons for failure in these trials may be that these studies have often included patients with all operable stages of NSCLC, irrespective of the local extent of the tumor, as opposed to the patients in our study, in whom preoperative radiation was used on IIIA/N2 tumors. It is possible that the benefit of preoperative radiation is seen only in patients with locally advanced disease, which does not extend to all other NSCLC patients.

The neoadjuvant treatment of NSCLC is a vast subject of interest. The debate on radiotherapy and surgery is based on deciding between preoperative and postoperative radiotherapy. So far, most studies have focused on the value of postoperative radiotherapy and less on preoperative radiotherapy. Due to the fact that preoperative radiotherapy usually takes 4 weeks, and a further recover period of 2‐3 weeks, it is difficult for patients and surgeons to accept such a long wait for surgery.^32^ However, preoperative radiotherapy leads to reduced lesions and surgical complications. Our study retrospectively compared the effect of preoperative radiotherapy on the prognosis of patients with stage III N2 NSCLC, to that of those who had not undergone radiotherapy. To obtain a clearer picture, we adopted the PSM method to process the data. The results showed that both before and after PSM, PrORT was superior in the management of stage IIIA/N2 NSCLC tumors. In the COX regression analysis, we found that preoperative radiotherapy was an independent prognostic factor for both CSS and OS. In the subgroup analysis, the advantages of preoperative radiotherapy are more pronounced in patients with T3 tumors and highly differentiated tumors.

There are several limitations to our study. Firstly, it is a retrospective study which carries a greater chance of bias in comparison with a prospective study. Secondly, we could not separate “Intentional selected” and “Compromised” from the SEER database. Details on pre‐and postoperative lung function, comorbidities, costs, details of induction therapy, major morbidity, the radicality of surgery and in‐hospital duration were unavailable. The details of concurrent chemotherapy regimens were also unavailable for further analysis.

## 5 CONCLUSION

Preoperative radiation may improve the outcomes of resectable IIIA/N2 NSCLC patients. In IIIA/N2 NSCLC patients, particularly patients with T3 and highly differentiated tumors, clinicians should boldly apply preoperative radiotherapy, to improve the patients' survival.

## CONFLICT OF INTEREST

None declared.
